# Re-stricture after buccal mucosal graft urethroplasty: a systematic review and meta-analysis

**DOI:** 10.1590/acb403525

**Published:** 2025-03-31

**Authors:** Gustavo Bono Yoshikawa, Gabriella Giandotti Gomar, Giovanna Ceccatto Gadens, Beatriz França Zanetti Saes, Maria Eduarda Andrade Galiciolli, Meire Ellen Pereira, Quelen Iane Garlet, Cláudia Sirlene Oliveira

**Affiliations:** 1 Instituto de Pesquisa Pelé Pequeno Príncipe – Curitiba (PR) – Brazil.; 2 Faculdades Pequeno Príncipe – Curitiba (PR) – Brazil.; 3 Universidade Federal do Paraná – Programa de Pós-graduação em Farmacologia – Curitiba (PR) – Brazil.

**Keywords:** Diabetes Mellitus, Urethral Stricture, Penile Diseases

## Abstract

**Purpose:**

To determine risk factors for re-stricture after buccal mucosal graft urethroplasty (BMGU) through a systematic review and meta-analysis.

**Methods:**

Following PRISMA guidelines, we collected data from PubMed, Scientific Electronic Library Online (SciELO), and Web of Science databases. The eligibility criteria included studies with male patients over 18 years old with urethral stricture recurrence after BMGU.

**Results:**

We retrieved 646 papers from three electronic databases. Records that did not meet the eligibility criteria and duplicates were excluded, resulting in 14 papers (3,240 patients) that underwent qualitative analysis, from which nine papers were suitable for meta-analysis. The meta-analysis identified diabetes mellitus (relative risk – RR: 1.58 **[95% confidence interval – 95%CI 1.02–2.46];** *p* = 0.04), penile/peno-bulbar site (RR: 1.57 [95%CI 1.04–2.37]; *p* = 0.03), and stricture size higher than 7 cm (RR: 4.13 [95%CI 2.42–7.04]; *p* 0.00001) as a predictive factor of re-stricture.

**Conclusions:**

These findings may improve understanding the risk factors for this type of urethroplasty and help surgical decisions. For a more effective analysis, larger and better-distributed study groups and cohorts are needed in the future to clarify whether the combination of a previous disease and the urethroplasty etiology may impact a recurrence-free outcome after stricture correction.

## Introduction

Male urethral stricture is often associated with urethral lumen stricture and may be caused by fibrosis or inflammation^1^. There are several etiologies involved in its physiopathology, such as pelvic trauma, sexually transmitted diseases, lichen sclerosus, infections and inflammations, hypospadias, prostate cancer, radiotherapy, impacted urethral stones, prolonged use of a urinary catheter, and idiopathic and iatrogenic causes^2^^-^ 5. Moreover, the postoperative transurethral resection of the prostate, transvesical prostatectomy, or radical prostatectomy may also cause urethral stricture^6^. Stricture can affect any urethra segment, such as the urethra prostatic, membranous, bulbar, and penile, or even the navicular fossa or bladder neck. Finally, strictures can be annular, short or long in extension, and may have some degree of inflammation^1^.

Treatment for this condition aims to restore the urinary flow and improve the patient’s quality of life. Several surgical techniques are used in stricture therapy, but the technique of choice depends on the size and complexity of the stricture. Internal urethrotomy is often used in annular and short strictures^1^^,^ 3. On the other hand, segmental urethrectomy is more effective in strictures shorter than 2 cm and/or with severe fibrosis. Furthermore, graft urethroplasty is the technique applied in more complex strictures.

The tissue used as the gold standard for graft urethroplasty is buccal mucosa^7^. The one stage buccal mucosal graft urethroplasty (BMGU) has a high overall success rate; Shalkamy et al.^5^ demonstrates an 87.2% success rate in their series. The buccal mucosal graft is preferable to the skin, bladder epithelium, tunica vaginalis, or small intestine mucosa grafts because it is more consistent in tissue thickness and composition. Additionally, this tissue has ideal features for this approach, such as moisture, vascularization, accessibility, and high levels of elastin, and it lacks hair or attachments^3^^,^ 7.

Success rates of long-term correction (average of 55 to 65.4 months) of a stricture using buccal mucosal grafts vary from 73 to 93.3%^8^^–^ 10. Stricture recurrence is associated with risk factors such as obesity, smoking, alcohol consumption, chronic diseases, age, physical status, American Society of Anesthesiologists (ASA) score, penile stricture location and length, etiology, previous surgery (due to the passage of surgical instrumentals through the urethra as transurethral resection prostate), and prior uretroplasty^5^^,^ 10 ^,^ 11. On the other hand, risk factors for re-stricture include fibrosis and ischemia in the stricture area^5^.

As both etiology and risk factors for re-stricture are not well explored and understood in the literature, the surgical technique and graft are chosen by the surgical team after analyzing each patient’s peculiarities and clinical characteristics following studies and practical guidelines on the subject^4^. There are some classifications of urethral strictures, such as scales and scores, that aim to establish a prognosis; unfortunately, these are not yet routinely used in scientific publications or in clinical practice. U-Score and MU-Score urethral stricture scores are currently validated. Eswara et al.^12^ simplified the U-Score with the variables size, penile or bulbar location, number of strictures and etiology. Shrivastava et al.^13^ demonstrated in their work a new score using size, etiology, comorbidities, previous interventions, location, indicating its better correlation with re-stricture after urethroplasty. In this surgical scenario, it is necessary to have a more solid scientific base to guide the medical decision toward a higher probability of urethroplasty success.

Accordingly, the present study aimed to compile and discuss putative risk factors involved in urethral re-stricture after BMGU through a systematic review and meta-analysis.

## Methods

### Study protocol

This systematic review was registered in PROSPERO (#CRD42021275041) and followed the Preferred Reporting Items for Systematic Reviews and Meta-Analyses (PRISMA) guideline^14^. The guiding question followed the acronym PICO: P = population, I = intervention, C = comparison, and O = outcomes^15^. In this case, P = male patients with urethral stricture; I = urethral stricture treated with BMGU; C = no re-stricture; and O = factors associated with urethral re-stricture after BMGU.

### Eligibility criteria

The selected studies followed the criteria:

- Studies with male patients over 18 years old with urethral stricture recurrence after BMGU;
- Cohort, case control, or cross-sectional studies;
- Studies published in English.

On the other hand, studies were excluded whether:

- Buccal mucosal graft procedures other than urethroplasty were evaluated;
- Urethroplasty with grafts other than the buccal mucosa was performed.

### Search strategy

The studies were retrieved during March 2022 in the following electronic databases: PubMed, Scientific Electronic Library Online (SciELO), and Web of Science. An update search was carried out in January 2024. The search strategies were conducted using the Boolean operators “AND” and “OR” as follows “urethral stricture” OR “urethroplasty” OR “urethral reconstruction” OR “penile strictures” OR “repair urethral” OR “urethral transection”, “reconstructive surgical procedures” OR “bulbar urethra” OR “anterior urethral strictures” AND “buccal graft” OR “buccal mucosal graft” OR “buccal mucosa graft” OR “autografts”.

### Study selection and data collection process

Initially, the records obtained from the above-mentioned search strategy were transferred to Mendeley Library (Elsevier BV, Amsterdam, The Netherlands) and the paper’s selection process was performed by four reviewers (GGG, BFZS, GCG, and MEAG). In the first stage of the review, each reviewer read the record title and abstract followed by classification under the eligibility criteria. Classification inconsistencies were solved by a reviewer pair (CSO and GBY). In the second stage of the review process, the potentially eligible studies were read to the full extent. Studies that met the inclusion criteria were maintained in the review process. After the studies’ selection, the data suitable to this systematic review were organized in a spreadsheet using the software Excel version 2016.

### Statistical analysis

We performed quantitative analyses using etiology, stricture size, stricture location, previous smoking, diabetes mellitus diagnostic, or previous urethroplasty as dichotomous variables. Continuous data were collected for stricture length and patient’s age, and it was shown as mean ± standard deviation (SD). Meta-analysis was performed using a 95% confidence interval (95%CI) for the analyzed studies. We analyzed the dichotomous variables by the Mantel-Haenszel method and the continuous variables by the inverse variance method. For both types of variables, a random-effects model was applied. Results from random-effects models were reported as relative risks (RR) with 95%CI for the dichotomous variables. Data were weighted by the sample size of each study. Heterogeneity was assessed by the I-square (I^2^) index and ranked as: no heterogeneity (< 25%), mild heterogeneity (25–50%), moderate heterogeneity (50–75%), and high heterogeneity (> 75%)^16^. Additionally, funnel plots were used to detect study bias. The statistical significance level was set at *p* < 0.05. All analyses were performed using the software Review Manager version 5.4.1 (Cochrane collaboration).

### Quality and certainty of evidence assessment

The quality assessment was performed using the Risk of Bias in Non-randomized Studies – of Interventions (ROBINS-I) tool, recommended by Cochrane to evaluate the risk of bias among non-randomized studies. ROBINS-I has seven domains, which include bias due to/in:

- Confounding;
- Selection of participants into the study;
- Classification of interventions;
- Deviations from intended interventions;
- Missing data;
- Measurement of outcomes;
- Selection of the reported result.

The studies were classified into low risk (low risk of bias for all domains), moderate risk (at least one domain classified as moderate risk), serious risk (at least one domain classified as serious risk of bias), critical risk of bias (at least one domain classified as the critical risk of bias)^17^.

A pair of researchers (MEP and CSO) independently evaluated the certainty of evidence for each risk factor (smoking, previous urethroplasty, diabetes mellitus, stricture cause – idiopathic, iatrogenic, and trauma, stricture place – bulbar and penile/peno-bulbar, and stricture size – < 7 cm and > 7 cm) and the outcome of interest (re-stricture) within the Grading of Recommendations, Assessment, Development and Evaluations (GRADE) framework^18^. We used the GRADEpro GDT online software (GRADEpro GDT: GRADEpro Guideline Development Tool; McMaster University and Evidence Prime, 2021) to summarize the evidence profile.

## Results

### Selection of papers

**Figure 1:**
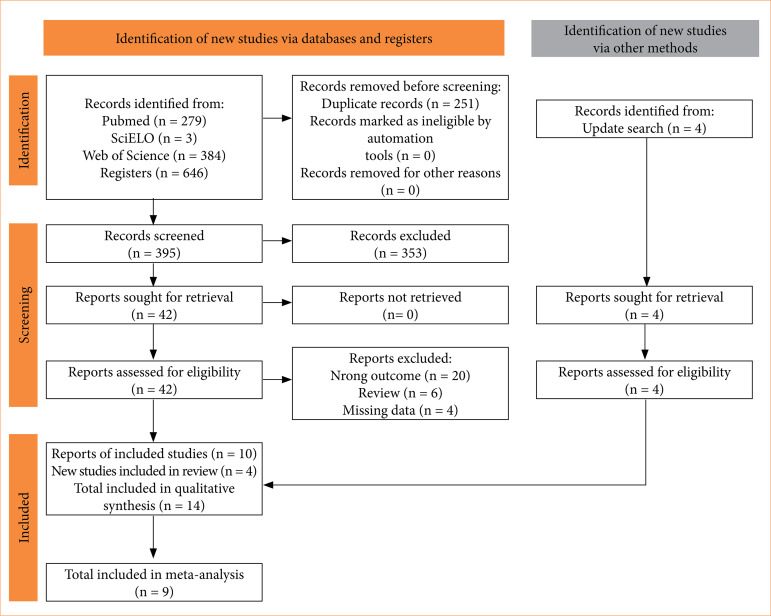
Preferred Reporting Items for Systematic Reviews and Meta-Analyses (PRISMA) flow diagram.

Source: Elaborated by the authors.

In this study, 646 papers were collected from three electronic databases. Duplicates accounted for 251 records; 385 studies were excluded because they did not meet the eligibility criteria. After the update search in 2024, four studies were included, resulting in 14 studies included in the qualitative analysis, from which nine were suitable for the meta-analysis (Fig. 1).

### Patients’ characteristics

The final 14 papers systematically selected for the qualitative analysis include studies conducted with patients from the continents of America (United States of America and Canada), Europe/Asia (Turkey, United Kingdom, Italy, Germany, and India), and Africa (Egypt) (Table 1). Together, these studies evaluated 3,240 male patients suffering from urethral stricture corrected by BMGU. Among them, 472 (15%) experienced stricture recurrence. The average age of the patients ranged from 29 to 56 years old and the average follow-up time for these patients ranged from 8 to 113 months. The average time to re-stricture fluctuated between seven and 52 months. The success rates of the BMGU reported in the systematically selected studies varied from 60.2–95%. The collected data indicated that 100% of the studies utilized one-stage anastomosis, while one study (7%) additionally employed a two-stage anastomosis.

**Table 1:** Main information of the systematically selected studies.

Authors	Country	Study design	Patient’s characteristics [N; Age (Mean ± SD)]	Success characterization	Average of follow-up (months)	Average time to re-stricture (months)	Success rate (%)	Anastomosis stage	Technique
Figler et al.^19^	United States of America	Retrospective	103; 40.8 ± 14.6	No need for endoscopic intervention, reconstruction revision, or cystostomy	35.7 ± 34.1	52.1 ± 62.9	82.0	One-stage anastomosis	Dorsal or ventral onlay (according to the surgeon’s choice)
Yalcinkaya et al.^20^	Turkey	Retrospective	40; N.I.	No need for intervention (including dilation) and when at six months uroflowmetry > 14 mL/s	43.4	N.I.	70.0	One-stage anastomosis	Dorsal onlay
Grossgold et al.^21^	United States of America	Retrospective	Overall: 91; 41.6 No Leak: 60; 44.7 ± 15.5 Leak: 31; 47.5 ± 13.4	Absence of stricture after urethrocystoscopy	Overall: 11.0 No Leak: 8.8 ± 24.9 Leak: 15.5 ± 24.9	N.I.	Overall: 83.5 No Leak: 85.0 Leak: 80.6	One-stage anastomosis	Dorsal (40 patients) and ventral (51 patients) onlay
Spilotros et al.^22^	United Kingdom	Retrospective	128; 42.8	N.I.	45.0	25.4	81.0	One-stage anastomosis (64 patients, bulbar); Two-stage anastomosis (61 patients, penile); Stricturotomy (3 patients, bulbar)	Dorsal onlay
Vetterlein et al.^23^	Germany	Retrospective	Overall: 534; 52.3 ± 16.6 Initial BMGU: 436; 52.2 ± 16.9 Repeat BMGU: 64; 50.7 ± 16.6 Secondary BMGU: 34; 56.0 ± 13.4	No need for intervention (including dilation)	Overall: 33.0 Initial BMGU: 33.0 Repeat BMGU: 32.0 Secondary BMGU: 32.0	Overall: 8.0 Initial BMGU: 11.0 Repeat BMGU: 16.0 Secondary BMGU: 7.0	Initial BMGU: 87.4 Repeat BMGU: 87.5 Secondary BMGU: 70.6	One-stage anastomosis	Ventral onlay (stricture bulbar) and dorsal inlay (stricture peniana)
Kumar et al.^24^	India	Retrospective	Non-CKD patients: = 130; 29.1 CKD patients: 90; 47.5	Uroflowmetry success: Qmax > 15 mL/s	Non-CKD patients: 23.3 CKD patients: 22.5	Non-CKD patients: 23.9 ± 16.5 CKD patients: 17.7 ± 12.1	Non-CKD patients: 95.4 CKD patients: 60.2	One-stage anastomosis	Ventral onlay
Meyer et al.^25^	Germany	Retrospective	517; N.I.	No need for intervention (including dilation)	32.0	N.I.	86.0	One-stage anastomosis	Ventral onlay (stricture bulbar) and dorsal inlay (stricture peniana)
Redmond et al.^26^	Canada	Retrospective	507; 45.4 ± 14.8	Absence of re-stricture defined as higher than 16 French on cystoscopy	78.9	N.I.	93.9	One-stage anastomosis	Dorsal onlay
Fuehner et al.^27^	Germany	Prospective	135; N.I.	No need for intervention (including probing and dilation)	21.2	N.I.	79.0	One-stage anastomosis	Ventral onlay (stricture bulbar) and dorsal inlay (stricture penile)
Shalkamy et al.^5^	Egypt	Retrospective	266; 37.7	No need for intervention (including urethral dilation)	49.8	21.6	87.2	One-stage anastomosis	Dorsal onlay
Güler^28^	Turkey	Retrospective	51; 54.8	Postoperative Qmax above 15 mL/s and > 17 French cystoscope easily passing the urethra	49.6 ± 8.0	N.I.	82.4	One-stage anastomosis	Dorsal onlay
Bandini et al.^29^	Italy	Prospective	575; 40 (30–53)	Absence of patient-reported bladder outlet obstruction symptoms with a normal urinary flow rate (> 10 mL/s)	113.5 (75.7–152.2)	N.I.	82.1	One-stage anastomosis	Ventral onlay
Güler^30^	Turkey	Retrospective	51; 51.6 ± 11.6	Urine flow > 15 mL/s and residual urine below 50 mL	30.7 ± 10.3	10.2 ± 5.1	86.3	One-stage anastomosis	
Sayedahmed et al.^31^	Germany	Prospective	22; 53.6 ± 11.4	Less than > 17 French cystoscope passing the urethra	38.6 ± 9.6	33.0 ± 12.5	86.4	One-stage anastomosis	

N.I.: not informed; SD: standard deviation; BMGU: buccal mucosal graft urethroplasty; CKD: chronic kidney disease; Qmax: maximum urinary flow. Source: Elaborated by the authors.

Furthermore, the dorsal technique was selected in 92.8% of the studies, while the ventral technique was utilized in 50% (Table 1). It is important to highlight that some studies explored multiple surgical techniques. Meanwhile, the factors that corroborate the procedure’s success are not consistent in the analyzed studies.

### Risk factors for re-stricture

The risk factors for re-stricture after BMGU were detected in ten out of the 14 systematically selected papers (Table 2). The main risk factors addressed as predictive of re-stricture were age, stricture length, and previous urethroplasty. However, it is worth highlighting that the statistical methods used to identify the risk factors were heterogeneous across studies, which may hinder a proper conclusion.

**Figure 2:**
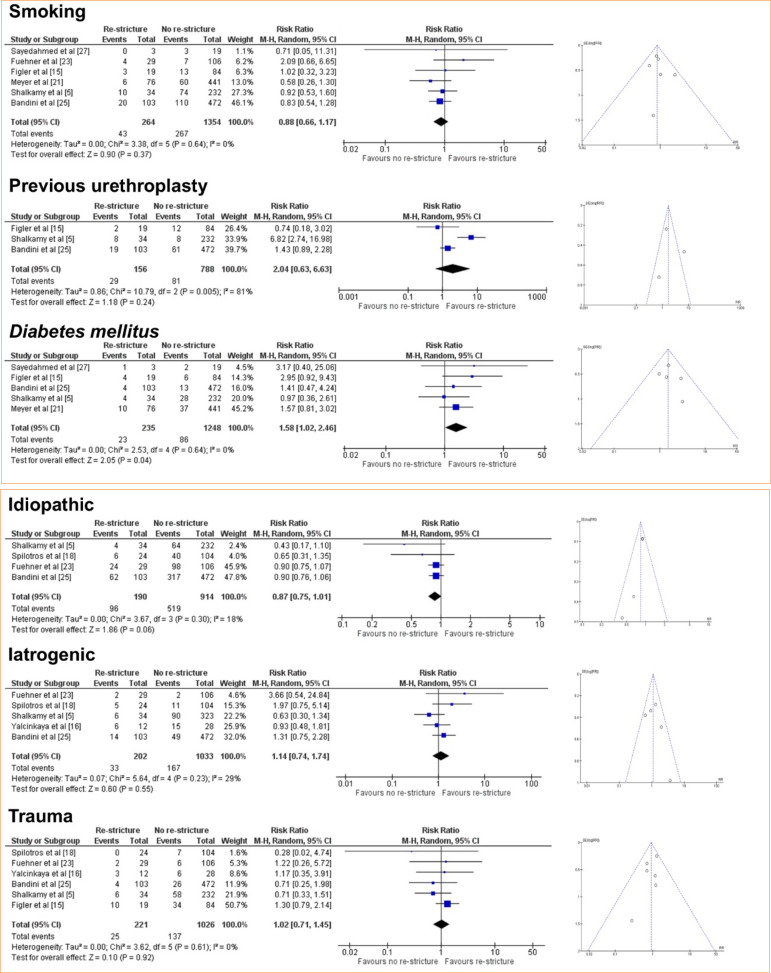
Forest and funnel plot of the risk ratio for re-stricture after buccal mucosal graft urethroplasty (BMGU) in smoking, diabetic patients, or patients that experienced previous urethroplasty, and stricture causes (idiopathic, iatrogenic, or trauma) as risk ratio for re-stricture after BMGU.

Source: Elaborated by the authors.

**Figure 3:**
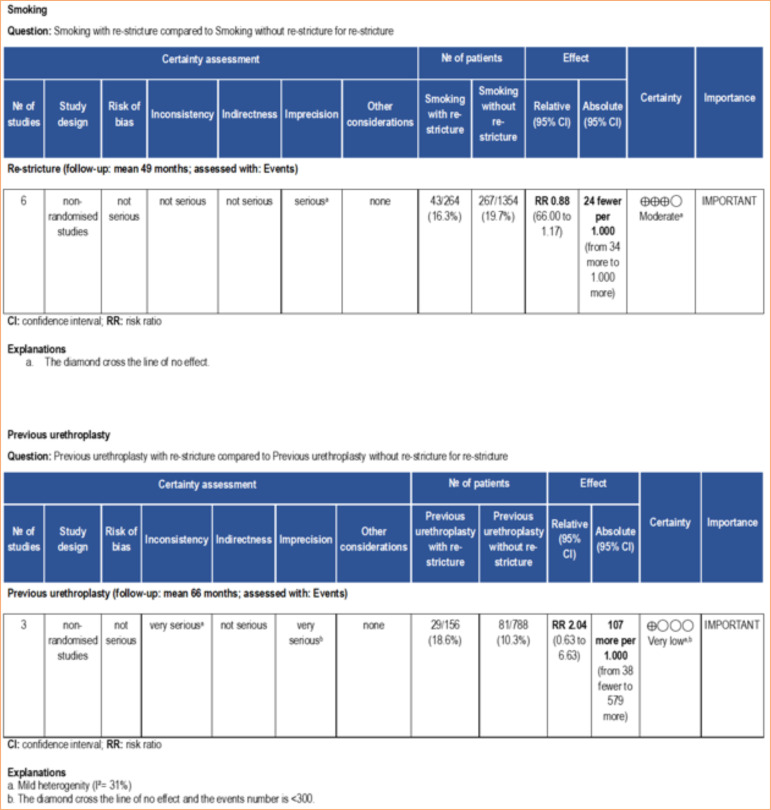
Certainty of evidence assessed using the GRADE framework. Certainty may be downgraded for risk of bias, inconsistency, indirectness, imprecision, or publication bias.

**Figure 4:**
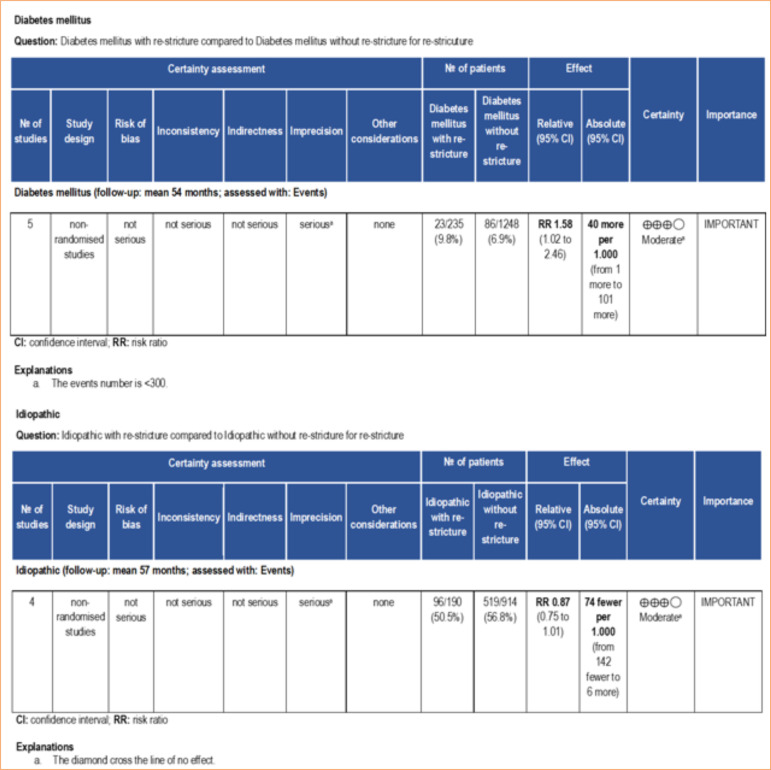
Certainty of evidence assessed using the GRADE framework. Certainty may be downgraded for risk of bias, inconsistency, indirectness, imprecision, or publication bias.

**Figure 5:**
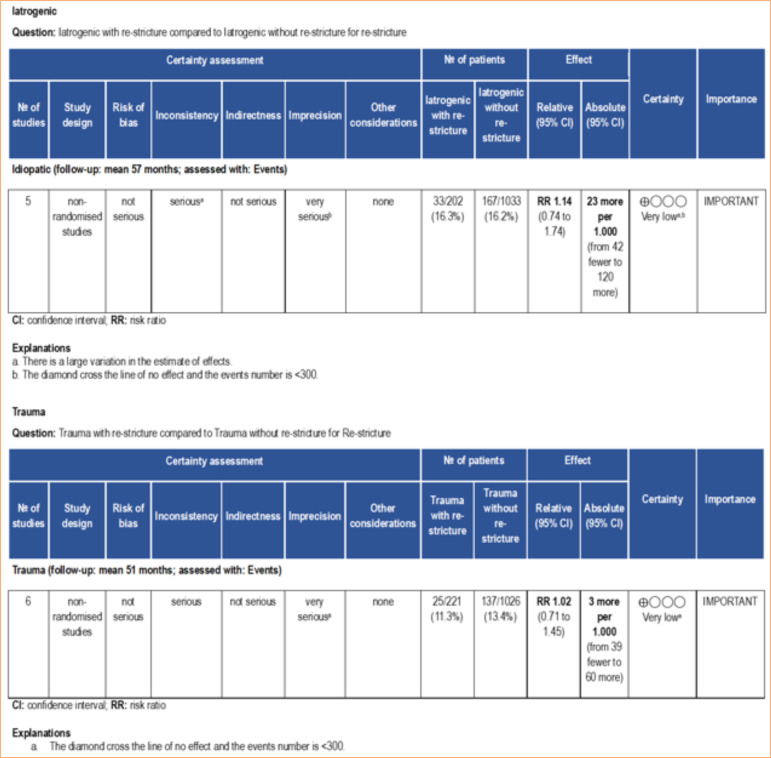
Certainty of evidence assessed using the GRADE framework. Certainty may be downgraded for risk of bias, inconsistency, indirectness, imprecision, or publication bias.

In this regard, the meta-analysis is a statistical tool that provides a more reliable data interpretation. In our analysis, smoking and previous urethroplasty, factors identified by some studies as predictive of re-stricture, were not found to be significant risk factors when meta-analyzed (Fig. 2). According to the GRADE framework, the certainty of evidence for smoking was classified as moderate, whereas for previous urethroplasty it was very low (Fig. 3). The very low certainty of the evidence for previous urethroplasty was primarily due to heterogeneity (I^2^ = 81%) and a low number of events (< 300). On the other hand, the meta-analysis revealed diabetes mellitus high (Fig. 2) as a predictive factor to re-stricture after BMGU (RR: 1.58 [95%CI 1.02 2.46]; *p* = 0.04). The GRADE framework classified the certainty of evidence for diabetes mellitus as moderate due to the low, number of events (< 300; Fig. 4). Moreover, re-stricture may occur regardless of the stricture cause (idiopathic, iatrogenic, and trauma) as no significant outcome was observed in the meta-analysis (Fig. 2). The certainty of evidence, according to the GRADE framework, was moderate for idiopathic causes (Fig. 4) and very low for iatrogenic and trauma causes (Fig 5). The very low certainty of evidence was primarily due to the low number of events (< 300).

**Table 2:** Significant risk factors for re-stricture after buccal mucosal graft urethroplasty (BMGU) pointed out by the systematically selected papers.

Authors	Risk factors	Statistical test
Yalcinkaya et al.^20^	Stricture length: > 7 cm Stricture site: panurethral, penile, penobulbar and bulbar	Pearson χ^2^ test and Fisher’s exact test
Grossgold et al.^21^	Leak length and width at initial postoperative urethrography	Kaplan-Meier analysis
Spilotros et al.^22^	Age: > 31 years old Etiology: Balanitis xerotica obliterans Stricture length: > 4.1 cm Stricture site: penile/penobulbar	t-Test and/or χ^2^ test
Vetterlein et al.^23^	Age: cut off age not informed American Society of Anesthesiology score: 3-4	Univariate Cox regression analyses
	Secondary BMGU	Kaplan-Meier analysis and Multivariate Cox regression analysis
Kumar et al.^24^	Stricture length: > 2 cm regardless estimated Glomerular filtration rate: < or > 60 mL/min	Fisher’s exact test
	Age: cut off not informed Previous disease: chronic kidney disease	Multivariate logistic regression analysis
Meyer et al.^25^	Age: cut off not informed American Society of Anesthesiology score: 3 Previous disease: coronary artery disease and hypertension Previous anticoagulant use	Univariable Cox regression analysis
Redmond et al.^26^	Comorbidity: not specified Stricture length: cut off not informed Augmented anastomotic urethroplasty technique Etiology: iatrogenic	Univariable or multivariable Cox regression analysis
Shalkamy et al.^5^	Etiology: inflammatory Stricture length: > 4.5 cm Stricture site: penile Urethral plate width: > 7 mm Previous disease: prior urethroplasty	χ^2^ test or Fisher’s exact test. Univariate and multivariate Cox regression analysis
Güler^28^	Stricture length: > 5.9 cm	Multivariate logistic regression analysis
Bandini et al.^29^	Stricture length: ≥ 5 cm	Univariate and multivariate Cox regression analysis

Source: Elaborated by the authors.

⊕⊕⊕⊕: high; ⊕⊕⊕◯: moderate; ⊕⊕◯◯: low; ⊕◯◯◯: very low. Source: Elaborated by the authors.

⊕⊕⊕⊕: high; ⊕⊕⊕◯: moderate; ⊕⊕◯◯: low; ⊕◯◯◯: very low. Source: Elaborated by the authors.

⊕⊕⊕⊕: high; ⊕⊕⊕◯: moderate; ⊕⊕◯◯: low; ⊕◯◯◯: very low Source: Elaborated by the authors.

**Figure 6:**
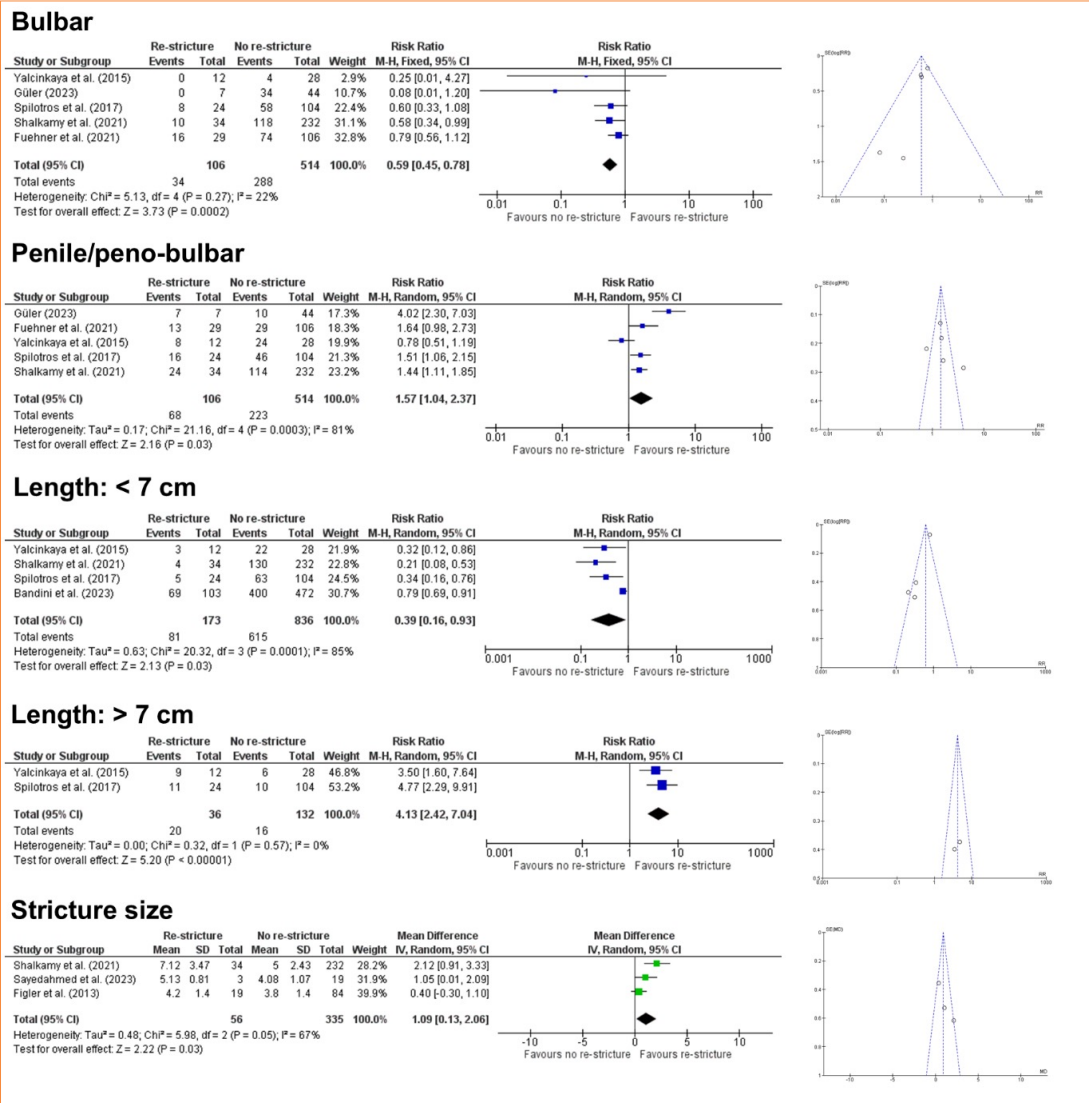
Forest and funnel plot for the stricture site (bulbar, penile/peno-bulbar) and stricture length (< 7 cm or > 7 cm) as risk ratio for re-stricture after buccal mucosal graft urethroplasty.

**Figure 7:**
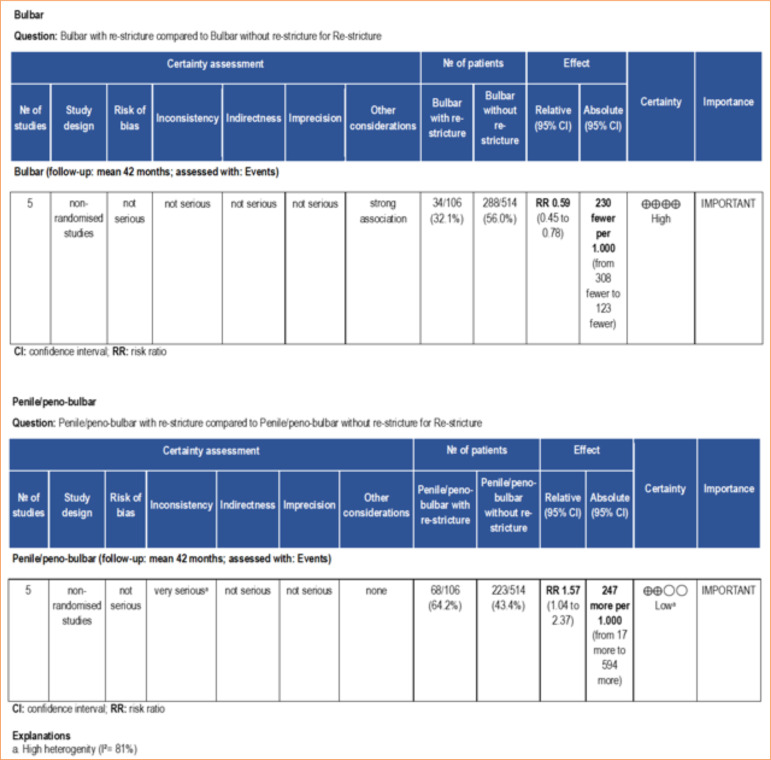
Certainty of evidence assessed using the GRADE framework. Certainty may be downgraded for risk of bias, inconsistency, indirectness, imprecision, or publication bias.

In its turn, the stricture site was pointed as a risk factor for re-stricture. The meta-analysis revealed that BMGU in the bulbar site has a low probability of evolving to re-stricture (RR: 0.64 [95%CI 0.46–0.90]; p = 0.01), while BMGU in the penile/peno-bulbar predicts a high risk to re-stricture (RR: 1.57 [95%CI 1.04–2.37]; p = 0.03) (Fig. 6). The certainty of evidence, according to the GRADE framework, was high for bulbar site and low for penile/peno-bulbar site (Fig. 7). The low certainty of evidence was primarily due to the high heterogeneity (I2 = 81%).

⊕⊕⊕⊕: high; ⊕⊕⊕◯: moderate; ⊕⊕◯◯: low; ⊕◯◯◯: very low. Source: Elaborated by the authors.

⊕⊕⊕⊕: high; ⊕⊕⊕◯: moderate; ⊕⊕◯◯: low; ⊕◯◯◯: very low. Source: Elaborated by the authors.

**Figure 8:**
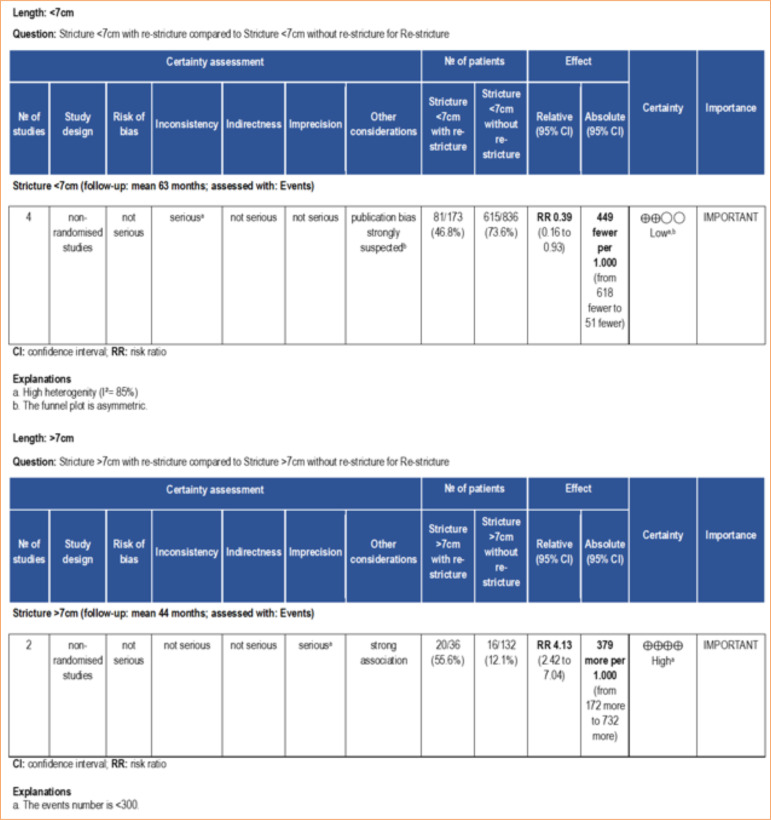
Certainty of evidence assessed using the GRADE framework. Certainty may be downgraded for risk of bias, inconsistency, indirectness, imprecision, or publication bias.

A similar pattern was observed when the stricture length was evaluated as a risk factor for re-stricture. The meta-analysis showed that strictures measuring lower than 7 cm predict a low risk of re-stricture (RR: 0.39 [95%CI 0.16–0.93]; *p* = 0.03). Accordingly, a stricture size higher than 7 cm predicts a high risk of re-stricture (RR: 4.13 [95%CI 2.42–7.04]; *p* < 0.00001) (Fig. 6). According to the GRADE framework, the certainty of the evidence for stricture size higher than 7 cm was classified as high, whereas for stricture size lower than 7 cm it was low (Fig. 8). The low certainty of evidence for stricture size lower than 7 cm was primarily due to high heterogeneity (I2 = 85%) and asymmetry of the funnel plot.

⊕⊕⊕⊕: high; ⊕⊕⊕◯: moderate; ⊕⊕◯◯: low; ⊕◯◯◯: very low. Source: Elaborated by the authors.

**Figure 9:**
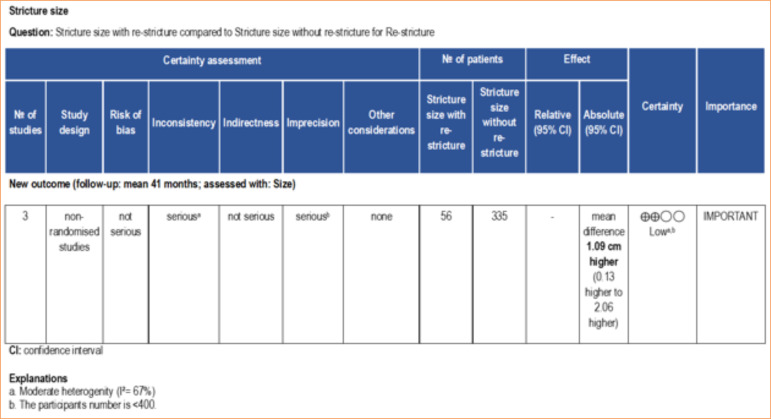
Certainty of evidence assessed using the GRADE framework. Certainty may be downgraded for risk of bias, inconsistency, indirectness, imprecision, or publication bias.

**Figure 10:**
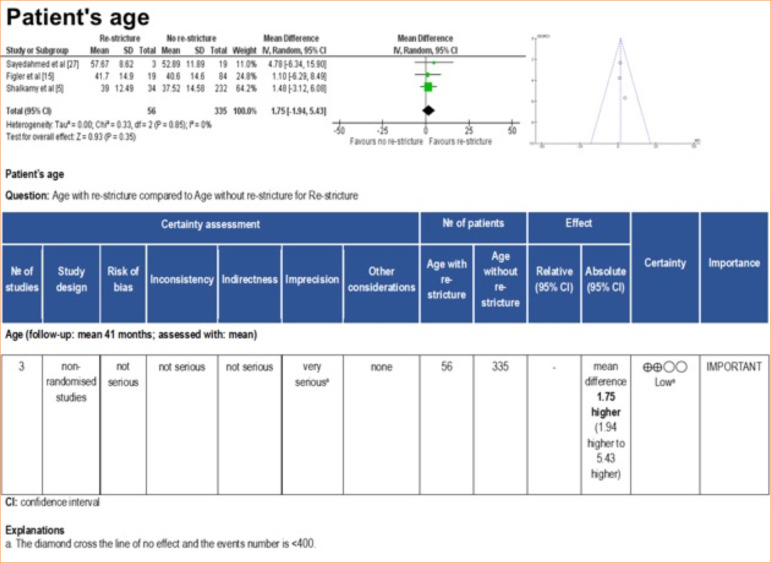
Forest plot, funnel plot, and GRADE framework for patients’ age with or without re-stricture after buccal mucosal graft urethroplasty.

Source: Elaborated by the authors.

Corroborating this finding, the meta-analysis revealed that the stricture size was slightly larger in patients who experienced re-stricture compared to those who did not suffer from this complication after BMGU (mean difference: 1.09 [95%CI: 0.13–2.06]; *p* = 0.03) (Fig. 6). The certainty of evidence, according to the GRADE framework, was low (Fig. 9) due to the moderate heterogeneity (I^2^ = 67%) and number of participants < 400. Age was not found to be a significant factor influencing the outcome of re-stricture (Fig. 10).

⊕⊕⊕⊕: high; ⊕⊕⊕◯: moderate; ⊕⊕◯◯: low; ⊕◯◯◯: very low. Source: Elaborated by the authors.

### Quality assessment

**Figure 11:**
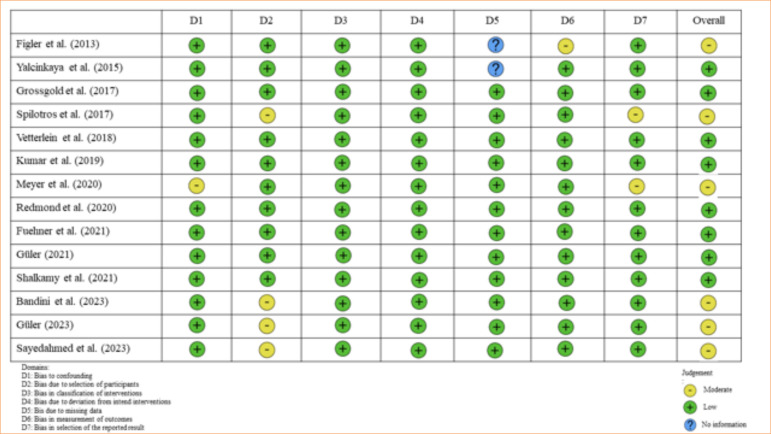
Risk of bias.

Source: Elaborated by the authors.

The quality assessment of the studies included in this systematic review detected a moderate risk of bias in 57% of the studies, while the remaining 43% displayed a low risk of bias (Fig. 11).

## Discussion

The first record of buccal mucosa used as the tissue of choice for urethroplasty dates to 1941 by Humby, and it gained strength thereafter in 1992^32^. Currently, there is a wide diversity of studies proposing a classification of the urethroplasty procedure including the urethral area, etiology, age, and other risk factors to improve decision-making regarding the choice of the surgical technique applied. In this systematic review and meta-analysis, we sought a better understanding of the interpretation of such data.

Open reconstructive surgery for urethral strictures has significantly improved in quality, safety, and success rates^33^. Urethroplasty is considered the gold standard in the treatment of anterior urethral strictures larger than 2 cm, as endoscopic treatments have practical limitations and low efficiency, with success rates potentially as low as 20%, compared to up to 80% for urethroplasty^4^. Reported procedure success using buccal mucosal graft reaches success rates up to 74% over ten years^9^. In the analyzed studies, a follow-up of at least seven months after the surgical procedure was deemed sufficient to provide a realistic perspective of the surgical outcome (procedure success or re-stricture).

Previous urethroplasty was considered a predictor for the recurrence of urethral stricture^5^. In the present analysis, there were no adverse effects from a previous urethroplasty on the re-stricture after a new intervention. However, on this subject, we only identified three eligible studies, which showed high heterogeneity between results and data variability. Thus, those studies cannot provide a proper conclusion on this matter. The final interpretation relies upon the fact that conflicting studies demonstrate the greater need for further BMGU studies to better determine whether the previous urethroplasty is a significant risk factor for re-stricture.

Failure in BMGU has been associated with comorbidities such as obesity and smoking^34^. Smoking associated with poor oral hygiene can affect wound healing and graft quality^5^. Indeed, it is established medical knowledge that diabetes and tobacco use increase microvascular damage, which, in turn, could impede and/or disrupt the healing processes after urethroplasty^10^^,^ 19. Conversely, a comprehensive analysis of the available data points out that smoking appears to play no role in the re-stricture development^19^. Accordingly, in our meta-analysis, we detected that smoking did not interfere with BMGU success, and this conclusion was based on a homogeneous (I^2^ = 0%) group of six studies. These findings may be explained due to smoking cessation at least 30 days before surgery, which could impact microvascularization. Despite being unsettling, this finding may help to better profile patients regarding their risk factors for re-stricture. Nevertheless, we encourage further studies to delve deeper into the role of smoking (including time smoking and smoking load) in surgical healing.

Another comorbidity that could interfere with tissue regeneration and healing is diabetes mellitus. Medical conditions such as diabetes mellitus are well known to cause microvascular damage, which in turn could disturb the healing process after the BMGU and induce stricture reformation^21^. Perioperative preparation of blood glucose levels can have a significant impact on the incidence of wound complications and, in turn, the recurrence of stenosis^5^. According to our meta-analysis diabetes mellitus is a significant risk factor for re-stricture. The impact of comorbidities, such as diabetes mellitus and overweight, has been widely studied, and the pathophysiology is highly correlated with blood flow, microvascular disease, and higher rates of dehiscence^35^.

One of the most controversial risk factors is stricture etiology. The inflammatory etiology is an independent factor of urethral re-stricture^5^^,^ 10. Conversely, this meta-analysis did not find significant prediction from the idiopathic, iatrogenic, or trauma etiologies. This result agrees with previous literature findings by the Spilotros et al.^22^ study, which found no association between these etiologies and re-stricture in a multivariate analysis. It is worth pointing out that, regarding the iatrogenic etiology, the meta-analysis detected a mild heterogeneity across studies (I^2^ = 29%), which may have biased the analysis conclusion. On the other hand, trauma and idiopathic causes data were homogeneous (I^2^ = 0 and 18%, respectively), which provided a more reliable inference. Taken together, these findings suggest that the etiology of re-stricture may not be a good predictor of either BMGU success or failure.

Regarding the stricture location, the literature is consistent. In another meta-analysis, penile urethral strictures corrected with BMGU were more prompted to failure compared to bulbar strictures corrections, reporting success rates of 77.6 versus 87.6%, respectively^36^. According to Spilotros et al.^22^, the penile urethral stricture is a predictor of re-stricture, as the authors report a recurrence rate of 25% in penile strictures compared to 12.1% in bulbar strictures. In Wang et al.’s^36^ study, the bulbar and penile urethroplasty had recurrence rates of 12.6 and 22.4%, respectively. In general, penile urethroplasty is a challenging procedure, with a risk of ischemia, infection, fistula, and re-stricture. Poor results are related to low vascularization in the distal urethra and the fact that the graft stretches in penile erections, compromising graft healing. Our meta-analysis retrieved homogeneous data (I^2^ = 22%) from studies with a low risk for re-stricture in procedures performed on bulbar stricture. Moreover, we detected a high risk for re-stricture in procedures performed on penile/peno-bulbar stricture; however, this finding is based on high-heterogeneity data (I^2^ = 81%). Therefore, our findings in this meta-analysis seemed to agree with the data available in the literature.

BMGU success rates are known to reduce when performed in strictures larger than 7 cm^20^, probably due to inadequate blood supply and the effect of inosculating blood vessels with the graft. In this meta-analysis, a low success rate in extensive stricture (>7 cm) was detected with statistical significance from two studies, while strictures smaller than 7 cm were pointed as a protective factor against re-stricture; however, this conclusion is based on high-heterogeneity data. The fact the low stricture size points to a low risk of re-stricture leads us to hypothesize that there could be a size-(re-stricture risk) relationship at play. Accordingly, a retrospective study with 604 patients found that patients with lichen sclerosis strictures and strictures larger than 5 cm displayed a higher chance of re-stricture^37^. In our meta-analysis, the average size was 7 cm to present statistical significance for restenosis, which could corroborate the finding on stricture size as a risk factor for re-stricture.

## Conclusion

In this meta-analysis, we detected that penile/peno-bulbar location and diabetes mellitus are significant predictors for re-stricture, while stricture located in the bulbar region offers a low risk for this outcome. Smoking, previous urethroplasty, and stricture cause (idiopathic, iatrogenic, trauma) were not identified as independent risk factors for stricture recurrence. On the other hand, the data regarding stricture size larger than 7 cm lacks statistical reliability, and the participation of these factors in BMGU outcomes should be approached with caution. These findings may improve the understanding of the risk factors for BMGU and help surgical decisions. However, this study had as a limitation the low number of studies suitable for meta-analysis. For a more effective analysis, larger and better-distributed study groups and cohorts are needed in the future to clarify whether the combination of a previous disease and the stricture etiology may impact a recurrence-free outcome after stricture correction with BMGU.

## Data Availability

All data sets were generated or analyzed in the current study.
